# What are the experiences of nurses delivering research studies in primary care?

**DOI:** 10.1017/S146342362300035X

**Published:** 2023-07-12

**Authors:** Azaria Ballintine, Rachel Potter

**Affiliations:** 1 University of Birmingham (Contingent Key Worker)/The Royal Wolverhampton NHS Trust (Research Nurse), UK; 2 Warwick Medical School, UK

**Keywords:** clinical trial, experiences, nurse, primary care, research, qualitative, research study

## Abstract

**Background:**

Clinical research provides evidence to underpin and inform advancements in the quality of care, services and treatments. Primary care research enables the general patient population access and opportunities to engage in research studies. Nurses play an integral role in supporting the delivery of primary care research, but there is limited understanding of nurses’ experiences of this role and how they can be supported to facilitate the delivery of research.

**Aim:**

To explore the experiences of nurses delivering research studies in primary care settings.

**Methods:**

We identified studies published between 2002 and June 2021 from key electronic databases. A two-level inclusion/exclusion and arbitration process was conducted based on study selection criteria. Data extraction and quality appraisal were performed simultaneously. Data were analysed in the form of a narrative synthesis.

**Findings:**

The key themes identified included: (1) what nurses value about primary care research and their motivations for study engagement, (2) the role of nurses in research, (3) working with research teams, (4) study training, (5) eligibility screening, data collection and study documentation, (6) nurse/participant dynamic, (7) gatekeeping, (8) relationships with colleagues and impact on recruitment, (9) time constraints and workload demands, and (10) health and safety.

**Conclusions:**

Nurses are integral to the delivery of research studies in primary care settings. The review highlights the importance of good communication by study teams, timely and study-specific training, and support from colleagues to enable nurses to effectively deliver research in primary care.

## Introduction

Primary care research increases opportunities for the general patient population to access studies and plays an important role in providing evidence to support improvements in patient care (Hyland and Clarke Moloney, 2016). In the United Kingdom, primary care research is mainly delivered in general practice but can include other primary care providers such as pharmacies and dental practices. Primary care services are strongly linked with wider community services such as community mental health, community nursing and residential and nursing homes who also contribute to the delivery of research (National Institute for Health Research (NIHR), 2021).

The NIHR Clinical Research Network (CRN) Primary Care Strategy outlines a vision to embed a coherent research theme within primary care through collaboration with the NIHR and wider strategic stakeholders, to encourage and support the delivery of high-quality research in a setting accessible to almost all the population (NIHR, 2022). However, delivering research in primary care has specific challenges, such as clinicians located across multiple sites and patients who access general practitioner (GP) services inconsistently (Graffy *et al.*, 2010). The absence of research infrastructure in many general practices can call for ingenuity on the part of practice staff to deliver studies effectively (Young *et al.*, 2009). High-quality research nurse support, prior to and during study delivery, is integral to the ability of GPs to support research activity (Gemzoe *et al.*, 2020).

In the United Kingdom, the day-to-day delivery of research in primary care is often nurse-led, with clinical oversight from a lead GP. Practice nurses may be an underutilised resource in the ambition to expand primary care research delivery (Shaw *et al.*, 2005). Due to the interdependency of primary care and community care services, a variety of nurses can be involved in the delivery of primary care-based research studies, including but not limited to practice nurses, community nurses, specialist nurses, hospice nurses and clinical research nurses. Nurses supporting research studies in primary care may be involved with recruiting patients, receiving informed consent, collecting data from patient records, conducting patient follow-up appointments and maintaining patient safety throughout study duration.

The aim of this review is to explore the experiences of nurses delivering research studies in primary care to understand how best to support nurses in this role.

## Methods

### Search strategy

We searched for studies published from 2002 to June 2021 from the following electronic databases: Ovid MEDLINE, Ebsco Cinahl, Proquest, Ovid PsycINFO, Web of Science; generic web searches (Google Scholar); grey literature (digital theses on UBIRA EThesis); and from reference lists of retrieved articles.

The search strategy used free text and medical subject headings; see Table 1. An initial scoping search used the SPIDER tool (sample, phenomenon of interest, design, evaluation and research type) to help define search terms.

**Table 1.** Search terms

Nurs* or ‘research nurs*’ or ‘clinical trial nurs*’ or ‘primary care nurs*’ or ‘community staff nurs*’ or ‘community nurs*’
AND ‘clinical trial’ OR ‘study’
AND (‘primary health care’ or ‘primary care research’ or ‘care home’ or ‘residential home’ or ‘General Practi*’ or ‘GP surgery’ or ‘family practi*’ or community) AND nursing research OR (research adj3 (deliver* OR workforce OR conduct)
AND (recruitment adj3 (patient OR participant OR subject)
AND ‘qualitative’ or ‘mixed method’ or ‘phenomenology’ or ‘grounded theory’

*Inclusion criteria:*

- Qualitative and mixed-method studies
- Focus groups, interviews and surveys
- Conducted in primary care and community settings
- Nurses involved in the delivery of research studies
- Published in English

*Exclusion criteria:*

- Quantitative studies
- Conference abstracts
- Research conducted in underdeveloped countries.

Box 1. Key recommendations

- Nurses should be asked rather than nominated to take part in research studies.
- Minimise the burden of work involved in delivering research studies.
- Study teams should provide regular communication and a recognised point of contact.
- Timely and study-specific training.
- Good communication, and support and understanding by colleagues.
- Protected/funded time for research activities when possible.
- Awareness of inadvertently acting as a gatekeeper to patients taking part in studies.

### Data management and screening

**Figure 1.**
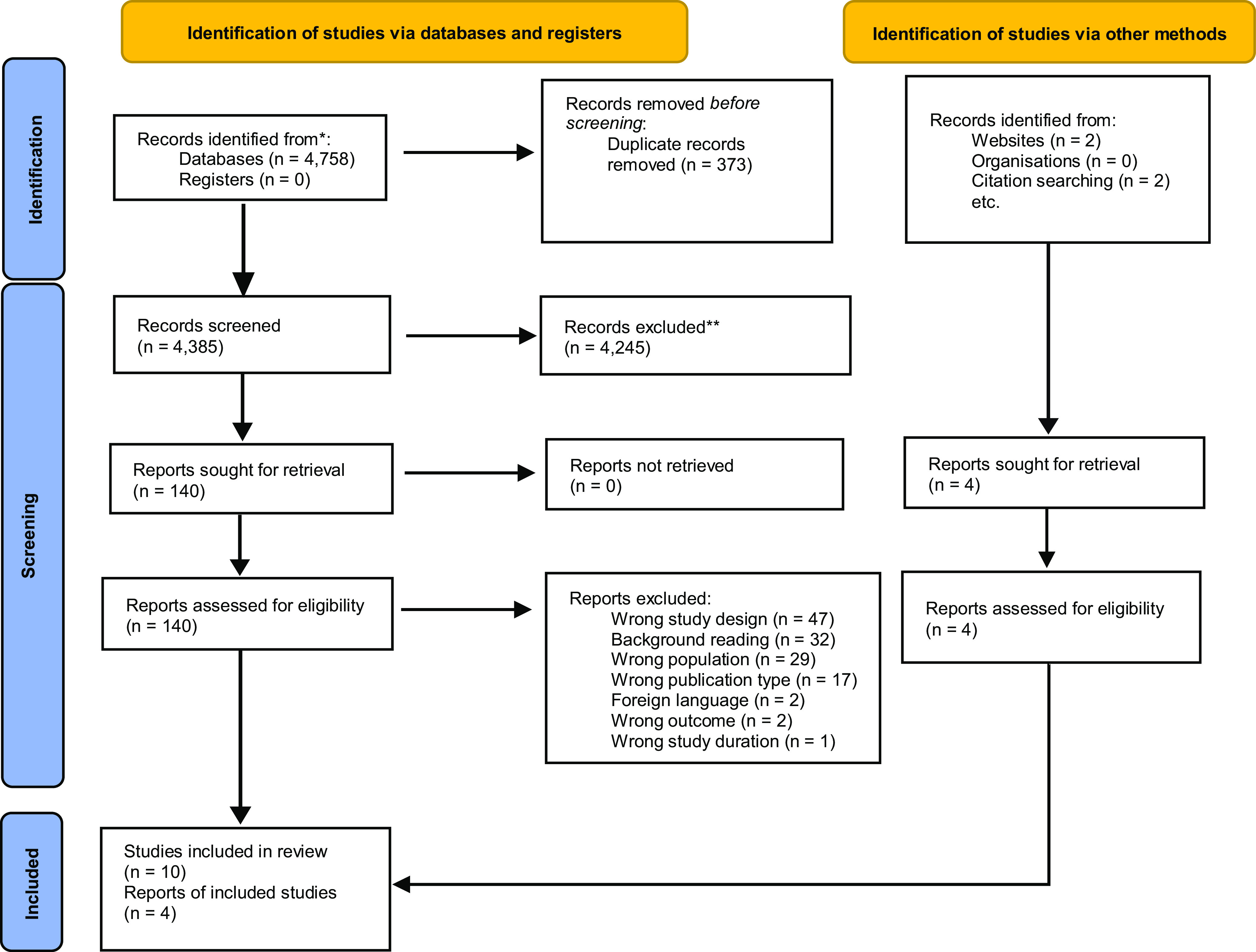
Flow diagram of included studies

Search outputs were uploaded to Endnote 20, and duplicates were removed. References were imported to Rayyan software for a two-level inclusion/exclusion and arbitration process. Titles were screened, and full copies of relevant papers were sought. The main reviewer (AB) screened records for inclusion and the second reviewer (RP) checked decisions to see if they concurred. Any disagreements were resolved by discussion. See Figure 1 for reasons for exclusion at full-text level.

### Data extraction and quality assessment

A customised data extraction spreadsheet was developed from an adapted version of the Joanna Briggs Institute (JBI) Data Extraction Tool for Qualitative Research (Aromataris and Munn, 2020). The main reviewer extracted data and the second reviewer checked the data extracted, with regular, ongoing communications to ensure agreement with decisions reached. The Critical Appraisals Skills Programme (CASP) checklist (Critical Appraisal Skills, 2018) was integrated into the data extraction spreadsheet to assess the quality of the studies and risk of bias.

### Analysis

We used a narrative synthesis to analyse and present our main findings. We considered a narrative synthesis appropriate to allow us to explore the similarities and differences between studies and provide a critical and objective analysis of the findings. Other methods of analysis could have been chosen, such as thematic synthesis, to identify commonality across studies. However, narrative synthesis was selected because the approach helps to clearly contextualise and characterise studies and can make heterogeneity between studies more apparent (Barnett-Page and Thomas, 2009).

## Results

A summary of the 14 studies identified for the review are presented in Table 2. The studies were published between 2002 and 2021 and from the United Kingdom (eight), United States (two), Australia (two) and Sweden (one), with geographical locations not specified in a systematic review (one). Studies were conducted in general practice (four), a nursing home (one), patients’ homes and/or clinic environments (five) or delivered across both primary and secondary care settings (four).

**Table 2.** Summary of studies included in the review

Author/year	Country	Setting	Participants	What is being researched	Summary of findings
Mentes and Tripp-Reimer (2002)	United States	Nursing home	Nurses, student nurses/nurse aides	Barriers and facilitators to conducting research in nursing homes	Barriers: resident characteristics; staff turnover, lack of leadership; administrative and organisational factors. Facilitators: nursing leader supportive of research, effective communication between NH staff and research team and well-coordinated data collection.
Shaw *et al.* (2005)	United Kingdom	General practice	Practice managers, general practitioners (GPs), practice nurses, research nurses, research coordinators	To explore the development of ‘research general practices’ in primary care.	The development of ‘research general practices’ was influenced by motivations to participate in research and by team roles and activity. GPs regarded their ‘research role’ as one of generating new knowledge, whereas nurses were motivated by the potential to improve care for their specific patient populations. ‘Research hierarchies’ were revealed in practices hosting research, with GPs often leading decision-making and nurses undertaking much of the groundwork. Lack of coordination across research team(s) appeared to hinder development, with shared decision-making helping to foster activity.
Potter *et al.* (2009)	United Kingdom	General practice	Practice nurses	Experiences of practice nurses recruiting to primary care research studies	There was generally positive engagement of practice nurses to participate in recruiting to a clinical trial. Dedicated time to recruit rather than financial incentives were considered important for nurses to recruit successfully. Nurses can unintentionally act as gatekeepers to recruitment, potentially restricting patients’ choice to take part in clinical trials.
Newall *et al.* (2009)	Australia	Community	District nurses	To explore the challenges and opportunities of a randomised controlled trial (RCT) conducted by two community nursing services	Key themes: being part of a trial, expectations versus the real RCT experience, benefits associated with implementing the trial, responses to the trial by other nurses not directly involved in the RCT, clients’ responses to the trial experience, challenges, strategies to refine research processes and further involvement in research.
Boase *et al.* (2012)	United Kingdom	General practice	Practice nurses	Experiences of practice nurses delivering a RCT in primary care	Time influenced nurses’ engagement with various aspects of the research, including the trial process and the delivery of the complex intervention. Nurses had to negotiate a range of competing loyalties due to interplay between their professional clinical role, their role in the research and within the practice teams.
Barr and Welch (2012)	Australia	Community	Nurse researchers	Workplace health and safety issues in nursing research	Researchers may perceive the level of risk of harm as lower than the actual or potential harm present in research projects. Nurse researchers do not consistently implement a risk assessment before or during research.
Fletcher *et al.* (2012)	N/A literature review	Primary and secondary care	GPs, hospital doctors, nurses, trainees, investigators and study staff	Synthesis of qualitative evidence related to clinicians’ attitudes towards recruiting RCTs	Communication of trial methods, education to remove misunderstanding of trial methods and reinforcement of the potential benefits of RCTs, both for clinicians and for their patients, were identified as future interventions aimed at improving clinicians’ recruitment activity.
Hange *et al.* (2015)	Sweden	Primary care, general practice	Nurses, practice managers, GPs and study nurses	Experiences of staff members participating in primary care research activities	Importance of including staff when designing clinical studies; information should be given continuously during the study and communication facilitated between different occupational groups working at the primary care centre.
Kyte *et al.* (2016)	United Kingdom	Local research networks and clinical trial units (primary and secondary care settings)	Research nurses, data managers/coordinators, trial managers and chief/principal investigators involved in clinical trials collecting PROMs	Opinions of researchers and trial personnel regarding the administration of patient-reported outcome measures in UK clinical trials	There are inconsistencies in the way PROMs are administered by trial staff. Such inconsistencies may reduce the quality of data and have the potential to introduce bias.
Lamb *et al.* (2016)	United Kingdom	Community	Community nurses	To identify the factors that impact upon the recruitment of participants to research studies in wound care from the community nurses’ perspective	Community nurses are positive about research activity and its potential benefits to patients and the clinical team. Research commitments are threatened by workload demands, which can influence timeliness of patient recruitment and lead to extra costs in terms of study extensions. Nurses consider additional factors beyond that of study eligibility criteria when determining patient suitability for a trial, and in thus doing, inadvertently introduce a pre-screening element to the recruitment process.
Morgan *et al.* (2017)	United States	In-person recruitment in clinical environments, telephone recruitment and community-based	Research coordinators, study nurses, professional recruiters and other professionals	How do study recruiters create positive relationships with potential participants?	Development of positive relationships with potential participants was credited to a number of behaviours: establishing a sense of personal connection (through reciprocal self-disclosures and other validating behaviours such as listening and expressing empathy); having repeated contact within a setting over a long or short period of time; demonstrating respect; exerting extra effort (or offering flexibility in the study process, usually in the form of appointment scheduling); and creating a sense of personal trust by being truthful.
Long *et al.* (2020)	United Kingdom	Acute and community care NHS trust settings	Research nurses	Research nurse perspectives on design and conduct of a RCT of wound care treatments	Involvement of research nurses (nurses specifically employed for research activities) in designing and developing processes for participant recruitment and retention, study conduct and intervention delivery is crucial to the successful conduct of a RCT.
Rose *et al.* (2021)	United Kingdom	Primary care	Health visitors (HVs) and community midwives	Exploration of factors that influence whether health visitors and community midwives invite patients to take part in research opportunities	Key barriers included time and resource constraints, perceived role conflict, competing priorities, and particularly for HVs, negative social influences from patients and researchers. Enablers included feeling confident to approach patients; positive influence from peers, managers and researchers; beliefs in the relevance of the research to healthcare and practice; and good knowledge about the study procedures, its rationale and the research topic.
McNiven *et al.* (2021)	United Kingdom	Primary and secondary care staff enrolled	Research nurses, midwives and allied health professionals	The experiences of research nurses, midwives and allied health professionals in relation to professional identity.	Nurses and allied health professionals experience considerable challenges as they enter and transition to a research delivery role, with implications for their sense of professional identity. A change in the work that they undertake and how they are (or perceive they are) viewed by others has implications for their sense of professional and individual identity.

Study methods included focus groups (three), individual interviews (five), surveys (two), mixed methods (three) and a systematic review (one).

Most studies (eight) included nurses as their sole participants: practice nurses (two), community nurses (two), nurses employed specifically to support the delivery of studies (one), health visitors and community midwives (one), nurses conducting their own research (one) and student nurses (one); herewith referred to collectively as nurses. The remaining six studies included nurses plus other health professionals, herewith referred to as ‘nurses and other staff’.

Nurses were either employed solely to work on research studies (five), or the nurses incorporated research delivery alongside their routine clinical work (eight); in the remaining study, this was unclear.

### Themes

We identified 10 key themes relating to the experience of nurses supporting research in primary care:

1. *What nurses value about primary care research and their motivations for engaging in research?*

Nurses appreciated the importance of primary care research and wanted to increase their knowledge and involvement in research processes (Hange *et al.*, 2015). Some nurses considered supporting research as essential to their professional role, believing that evidence-based research findings could enhance clinical practice and patient care (Rose *et al.*, 2021).

Motivation for taking part in research often centred on perceived improvements in patient care. Nurses referred to extended consultation times, access to new treatments and equipment, and the enjoyment they gain from additional patient contact (Shaw *et al.*, 2005). Nurses reported positive patient outcomes such as improved wound healing or helping patients come to terms with a disease (Newall *et al.*, 2009; Potter *et al.*, 2009).

Nurses attested the experience of taking part in research had positively changed their practice by providing an opportunity to reflect on their normal clinical approach (Boase *et al.*, 2012). Some nurses thought the status of their organisation would rise due to the credibility afforded from taking part in quality research, and that their participation in research could raise the profile of nursing (Newall *et al.*, 2009).

There was some evidence that how nurses felt about delivering research was affected by whether they were asked if they wanted to contribute to the delivery of a research study (Newall *et al.*, 2009). Nurses who were nominated to recruit patients to a study felt burdened, whereas nurses who were asked reported positive experiences of study involvement (Potter *et al.*, 2009).

1. *The role of nurses in research*

Not all nurses felt confident in their new role of delivering research studies and needing to acquire new knowledge and competencies (Hange *et al.*, 2015). Some nurses found autonomous working whilst supporting research studies less of a transition from a previous post which had involved independent working. Nurses and other staff acknowledged that skills from their previous employment (e.g., communication and phlebotomy) were transferrable and an asset to supporting trial delivery (McNiven *et al.*, 2021).

Nurses made decisions about multiple existing agendas in order to manage research delivery in a real-world setting (Boase *et al.*, 2012). Nurses found designation of roles during the study helpful, but they also found it challenging to combine clinical work with research (Hange *et al.*, 2015). Some nurses indicated that the research topic being addressed needed to be relevant to their roles and duties and identified the potential for role conflict (Rose *et al.*, 2021).

Nurses reported finding it challenging to take on a new role (research identity), encountering conflict between their roles, being a health professional with loyalty to patients and seeking to meet the demands of the practice, plus being part of the research team and striving to meet the obligations of a study (Boase *et al.*, 2012). McNiven *et al.* (2021) acknowledged that although a nurse employed specifically to do research may enter a clinical setting solely to conduct research-related duties (e.g., data collection and patient recruitment), they may be inclined to approach these obligations from a general nursing perspective. Nurses, therefore, need to be able to adapt to their new role and recognise that they are no longer working in the capacity of a member of the clinical team but are on site to support research.

1. *Working with research teams*

Nurses reported wanting to be regarded as collaborators in research that is clinically relevant to practice and to be offered the opportunity to contribute to study design to optimise recruitment and increase sense of ownership (Fletcher *et al.*, 2012; Hange *et al.*, 2015). Nurses who enjoyed participating in the planning and design stages of the study and helping to identify and resolve potential issues shared this viewpoint (Newall *et al.*, 2009).

Communication between staff delivering the study and the research team was noted to impact on study promotion, staff engagement and study recruitment. Nurses reported a lack of encouragement from research teams as a barrier to supporting research, and communicative and visible study teams as a motivator (Rose *et al.*, 2021). Nurses expressed reduced contact with researchers during trial recruitment resulted in lost opportunity to ask study-related questions, fewer recruits and decreased motivation (Hange *et al.*, 2015). Nurses considered it of great importance to establish a connection with the research team and valued having a point of contact (Lamb *et al.* 2016).

1. *Study Training*

The importance of assigning adequate time and resources for study training and study processes was identified by Boase *et al.* (2012). Rose *et al.* (2021) described how researchers should involve nurses in the design of training for a study and re-evaluate study training to ensure it continues to meet the needs of those for whom it is intended. Reducing the amount of time between receipt of study training and the commencement of recruitment could improve study engagement (Long *et al.*, 2020).

Training should cover study processes, recruitment, study rationale and the research topic (Rose *et al.*, 2021); information on methodology may also be beneficial (Fletcher *et al.*, 2012). Specialised research terminology used in the initial training session in the study by Boase *et al.* (2012) was unfamiliar to nurses and may have added to their anxiety. Repeats of study training (Hange *et al.*, 2015), additional booster sessions and mock recruitment exercises may all be beneficial (Mentes and Tripp-Reimer, 2002).

Kyte *et al.* (2016) highlighted several issues around training on patient-reported outcome measures (PROMs). Nurses felt they received little PROM training, and that additional training would enhance their ability to explain to participants why PROM data is collected and why it is important for a study. Nurses thought PROM training should include how to answer ambiguous questions and what to record when participants’ answers do not match available responses.

1. *Eligibility screening, data collection and study documentation*

Screening patients for study eligibility was more intensive than anticipated, and nurses struggled to find suitable patients (Long *et al.*, 2020). Nurses sometimes found eligibility criteria too restrictive as they excluded patients who most presented with the health complaint being studied (Newall *et al.*, 2009). Confusion amongst nurses and other staff about study eligibility criteria and which version of the protocol was being used led to one nurse feeling undermined when her initial decision to exclude patients was questioned by a colleague (Long *et al.*, 2020).

Having additional time to interview potential participants for inclusion could have been advantageous, and time constraints meant nurses were unable to ask enough questions about patients’ symptoms, reducing opportunities for inclusion (Hange *et al.*, 2015). Some nurses developed helpful strategies to promote research studies, such as notices about the study in clinic rooms and computer screen alerts (Potter *et al.*, 2009).

Instruction on data collection processes needs to be clear and in the most appropriate form for nurses to access. Clear guidance in the study protocol can help avoid differences in interpretation and inaccuracy of data collection (Long *et al.*, 2020; Kyte *et al.*, 2016).

Obtaining study data can be time-consuming, particularly in community settings when patients’ medical records are not readily to hand (Long *et al.*, 2020), with some nurses reporting to get fed up with data collection (Newall *et al.*, 2009). High staff turnover, inflexible staff work schedules and challenging study population characteristics (e.g., cognitively impaired) can hamper data collection efforts (Mentes and Tripp-Reimer, 2002).

The initial research information provided to practices should succinctly describe the study, and the study methodology should be easy to understand and convey to patients (Fletcher *et al.*, 2012). Terminology used in study documentation can be open to potential bias, for example, nurses regarded one participant’s information leaflet as emphasising the intervention more than the control (Long *et al.*, 2020). Study information should be comprehensive and accessible to equip nurses with the knowledge needed to answer patient queries, without requiring them to spend additional time reading about the research subject (Rose *et al.*, 2021).

Scripted protocols are a guided dialogue provided by study teams to recruiting staff to standardise their communications with patients. However, scripted protocols may result in less personal and more robotic communications (Morgan *et al.*, 2017). Nurses found using a scripted protocol formulaic, repetitive, uncomfortable and patronising towards patients (Boase *et al.*, 2012).

1. *Nurse/participant dynamic*

Nurses prioritised developing trust with potential participants, empowering patient decision-making around whether to take part in a study out of choice rather than obligation (Lamb *et al.*, 2016; Morgan *et al.*, 2017). Nurses thought patients may be less likely to take part in a study without the presence of an amicable relationship (Lamb *et al.*, 2016). Participants recommended taking part in the study to friends and family based on their perception of the nurse/staff member, rather than the study itself (Morgan *et al.*, 2017).

Nurses believed a good relationship with prospective participants could be developed, and disengaged participants may be disarmed, by adopting a highly polite manner, using formal forms of address, and showing appreciation by thanking individuals for giving up their time to engage in research. Nurses commonly went above and beyond to accommodate participants (e.g., maximising appointment flexibility and seeing late arrivals), which improved nurses’ ability to recruit and retain participants (Morgan *et al.*, 2017).

Nurses and other staff found it challenging to get across salient points about a study to patients, yet were aware their choice of language, and ease in communicating with patients with whom they identify (e.g., similar social class) could be influential (Fletcher *et al.*, 2012). Some likened explaining the process of randomisation to a sales pitch, or a description of the lottery, with winners and losers. McNiven *et al.* (2021) highlight that how a clinician conveys the patient information sheet to a participant (using vocabulary they understand) contributes to how well it is understood. The consequences of effective communication were demonstrated in the study by Newall *et al.* (2009) in which some nurses were surprised that patients were more tolerant of compression bandaging than they had anticipated and attributed this to better explanation of its efficacy.

Nurses considered it important to appreciate and address the patient’s own agendas before recruiting them as a study participant (Boase *et al.*, 2012). This was evident in the study by Newall *et al.* (2009) where resistance to study involvement was sometimes voiced by patients who thought participation may limit their freedoms and lengthen their district nurse visits.

1. *Gatekeeping*

Nurses acted as gatekeepers, not approaching all patients who met study eligibility criteria, but only those who they deemed suitable (Fletcher *et al.*, 2012; Rose *et al.* 2021). When assessing patient eligibility, some nurses introduced additional factors to include or exclude a patient (Lamb *et al.*, 2016). For example, nurses were more inclined to approach patients who demonstrated good communicability, motivation, enthusiasm, interest and a good nurse/patient relationship (Lamb, Backhouse and Adderley, 2016). Conversely, some nurses tended to select patients who were non-compliant with their treatment in the hope that the study may help them reconsider their outlook (Potter *et al.*, 2009). Nurses were dissuaded from inviting patients with frailty/poor health, impaired mental capacity, social issues (isolation or recent bereavement), environmental issues posing concern for nurse safety (Lamb *et al.*, 2016), a lot of care input or who had been on the nurse caseload for a long time because they thought patients would not like it (Potter *et al.*, 2009).

Fletcher *et al.* (2012) explored some of the reasons gatekeeping took place. Nurses were concerned that study invitation may affect their dynamic with patients and did not want to be perceived as pushing patients to take part. Nurses grappled with the potential risks/side effects posed to patients versus the wider population gain research produces. Nurses factored in the timing and emotional burden of research involvement for patients who are terminally ill or with a poor prognosis.

Although nurses may be well intentioned, this additional pre-screening element potentially creates sample bias, a loss of patient autonomy, and a loss of valuable data on a hidden population (for which the size and demographic are unknown to both the researcher and patient) and limits generalisability of research findings (Lamb *et al.*, 2016).

1. *Relationships with colleagues and their impact on recruitment*

Engaging colleagues in the research process can positively affect study delivery. Staff working collaboratively, with good communication and a shared research vision, can help to minimise resentment by non-study staff and promote patient recruitment.

It was important for participating practice teams to wholly adopt a shared research vision (Boase *et al.*, 2012) and for all nurses, not just those working on the study, to be kept updated on the research processes to minimise feelings of resentment or exclusion (Newall *et al.*, 2009). Facilitators to effective collaboration include sharing knowledge and experiences of good practice during study recruitment, joint working on study activities (recruitment and data collection), and the ability for nurses to be flexible, compatible and accommodating (Mentes and Tripp-Reimer, 2002). By implementing weekly progress reviews of trial recruitment, Newall *et al.* (2009) noted that this might lead to effectual collaborative working, information sharing and problem-solving. Challenges to collaborative working include time needed to liaise with other health professionals, unreliability of other health professionals to support research activity because of their clinical priorities (Long *et al.*, 2020), and the presence of hierarchical positions within GP practices, with a lack of collaborative decision-making (Shaw *et al.*, 2005).

Long *et al.* (2020) described how nurses spent a large proportion of time trying to raise the profile of a study by phoning and emailing trust staff and visiting clinical areas, yet engagement from colleagues to support the study was inconsistent. Nurses reported miscommunications with care home staff about sample collection, with staff ‘selectively hearing’ about trial obligations (Mentes and Tripp-Reimer, 2002).

Practice nurses reported feeling isolated working in a research capacity, with some being the only member of the team involved with the study. They reported experiencing resentment or concern from other nurses in the team who perceived clinical tasks as not prioritised due to research demands (Boase *et al.*, 2012). One nurse was concerned that colleagues regarded her as sitting and ‘doing nothing’ when attending to research obligations (Hange *et al.*, 2015).

1. *Time constraints and workload demands*

Time is a well-documented barrier to the ability to support the delivery of studies (Fletcher *et al.*, 2012) and was reported in 9 of the 14 studies. Research duties may not be prioritised over existing obligations of achieving service targets (Mentes and Tripp-Reimer, 2002; Fletcher *et al.*, 2012).

Boase *et al.* (2012) found that when practice nurses were not allocated protected time for study activities, this compounded pressure on both their clinical and research work. In research-naïve practices, the challenges of securing allocated protected research time, separate to clinical duties, created tensions in work relationships (Shaw *et al.*, 2005).

Funded protected research time for nurses and other staff may improve recruitment and enable detailed explanation of the study to participants (Fletcher *et al.*, 2012). Potter *et al.* (2009) acknowledged that despite fees being paid to support practices with recruitment, dedicated time for recruitment only featured at a few sites. Nurses who allocated dedicated time for patient recruitment were more successful at recruiting participants.

High workload, competing priorities and the unpredictability of recruitment made it challenging to resource the research study with nurse time (Newall *et al.*, 2009). High workload can result in insufficient time for nurses and other staff to perform research activities (Hange *et al.*, 2015). For nurses not solely delivering research studies, study duties (e.g., assessing eligibility and receiving informed consent) created additional work over and above their usual workload (Fletcher *et al.*, 2012). Some nurses felt research funding should cover them for protected time to approach potential participants about study participation, rather than conducting research on top of their existing workload (Rose *et al.*, 2021). Study commitments in addition to usual workload can overwhelm nurses, especially when they are particularly pressured (Mentes and Tripp-Reimer, 2002).

Research teams should minimise the burden of work for nurses delivering research (Newall *et al.*, 2009). If funded protected time cannot be achieved, then reduction of workload related to study recruitment is critical to improving study recruitment (Fletcher *et al.*, 2012). Administrative staff could reduce the amount of time nurses spent recruiting patients and arranging follow-up visits (Boase *et al.*, 2012).

1. *Health and safety concerns*

Barr and Welch (2012) explored workplace health and safety issues for nurses conducting research in the community. Most participants perceived their risk of harm to be minimal and tended to only complete perfunctory risk assessments that they saw as a requirement for their employers rather than for their own safety. Yet participants shared examples of their experiences of health and safety issues that arose when delivering studies including lone working risks, being stalked by a research participant and concerns for the welfare of others. The authors recommended that nurses would benefit from more understanding of the purpose of risk assessments and tips to disengage from researcher–participant relationships.

## Discussion

Nurses placed varying degrees of importance on conducting healthcare research influenced by: whether they considered research an incumbent part of their role; if they had been asked or nominated to support a research study; whether their contributions were adequately acknowledged; or whether the study covered a subject area they were interested in. The latter point supports findings by Rait *et al.* (2002) who recognised that practice nurses were keen to participate in research relevant to their practice population, and Davies *et al.* (2002) who observed that practice nurses doing their own research opt to study long-term health problems high in prevalence in their local patient population.

Barriers to nurses engaging in research included insufficient time, lack of support from colleagues and poor access to higher education resources (Davies *et al.*, 2002). Motivators included: perceived improvements to patient care, patient outcomes and clinical practice; personal benefit; career development; and raising the calibre of one’s organisation or nursing discipline.

The review highlighted the need for nurses to be involved in study design and study training, helping to identify and mitigate potential issues and shore up the efficient running of a study. Training can help nurses develop skills to face the challenges of study delivery, ensuring safe and ethical care is provided to research participants and high-quality data are collected (Hernon *et al.*, 2020). Study training should address the research topic, the rationale for conducting the study, study processes and recruitment. Ideally, training should be study-specific and practice-based (Rait *et al.*, 2002). Staff should receive training that includes some explanation of the rationale behind aspects of the protocol (e.g., inclusion/exclusion criteria) and the consequences of misconduct on the study and research objectives (True *et al.*, 2011). Specific training on PROM assessment methods, which are frequently used data collection methods, was also considered important.

Whether nurses delivered the research as their sole role, or in addition to an existing clinical role, impacted on the workload demands placed on nurses. Young *et al.* (2009) describe how ever-increasing workloads, insufficient support from medical colleagues and competing demands featured as major obstacles for nurses in research active general practices. Competing and organisational pressures can make it difficult to deliver research in primary care (Gaglio *et al.*, 2006), with clinical commitments posing the greatest barrier to research participation (Rait *et al.*, 2002). The literature review found that measures to reduce nurse workload have favourable outcomes on study delivery, and that nurses who allocated protected time were more successful with recruitment.

Hernon *et al.* (2020) described how clinical research nurses experienced isolation and a lack of understanding from colleagues about their role, creating difficulties for study recruitment. The review identified that fostering a good relationship with the wider working team can help nurses deliver research studies efficiently and minimise feelings of isolation and resentment. Regular and supportive dialogue between study teams and nurses bolsters study promotion, staff engagement and recruitment.

Nurses may find changing to a research role challenging and draw solace from existing, transferable nursing skills and experiences. Spilsbury *et al.* (2008) specify obstacles nurses associate with the role transition, namely lack of confidence, role conflict and difficulties encouraging clinical nursing staff to comply with study protocols, whilst maintaining their own motivation.

The review highlights the conflict nurses encounter being both a clinician and staff member supporting the delivery of research. Nurses were internally juxtaposed with being a patient advocate, whilst adhering to a study protocol. Tinkler *et al.* (2018) acknowledge the ethical issues nurses face when they feel patients may not truly understand the implications of taking part in a research study. Duncan *et al.* (2009) describe the tensions research staff encounter between encouraging open disclosures from research participants in qualitative interviews and acting on shared information in the best interest of the participant. Nurses felt especially pressured and like reluctant salespeople when working on industry-funded studies where recruitment targets were high (Tinkler *et al.*, 2018).

The review identified some key factors that can affect data collection by nurses: staff designation to the task; study eligibility criteria; characteristics of the study population; accessibility of study data; and whether guidance on data collection processes was clear. It was important for nurses that study documentation was comprehensive and easily understood, and scripted protocols were off-putting for some nurses. Data collection was more challenging, and health and safety risks were more notable for community nurses.

The professional regulatory body for nurses in the United Kingdom, the Nursing and Midwifery Council (NMC), stipulates that nurses should practice in accordance with best available evidence and collect, treat and store all research findings befittingly (NMC, 2018). Nurse-led research and studies delivered by nurses can propel change. Evidence procured through research moulds the profession of nursing, informing policy and professional decision-making. Cultivating an environment for nurses to flourish in leading, and to participate in and deliver research for patient benefit is a key objective outlined in the chief nursing officer (CNO) for England’s strategic plan for research (National Health Service, 2021). Exploring nurses’ experience of delivering research studies in primary care is an important step to understanding how best to support nurses in contributing to the CNO’s strategic plan.

### Study limitations

A possible limitation of this review is that it only includes studies written in English, potentially omitting relevant studies and contributions to the subject area.

Our inclusion criteria included publications from the last 20 years, a period of significant changes to nursing roles and the healthcare system. The earlier publications could, therefore, seem less relevant. However, some of the issues identified in the earlier publications remain pertinent today and help to reinforce the relevance of the review. Evidence from the earlier publications exploring the historic experiences of nurses supporting and delivering research studies in primary care should help inform contemporary work moving forward.

## Conclusion

Nurses are integral to the delivery of primary care research studies. This review explored the experiences of nurses delivering research studies in primary care and identified potential challenges and facilitators to effective study delivery. The review highlighted the importance of good communication by study teams, timely and study-specific training, and support and understanding from colleagues. Nurses value their relationships with patients and the benefits that research participation can achieve, but some nurses may inadvertently introduce bias when considering patient suitability for trial involvement. Offering nurses protected time to conduct research tasks improves trial recruitment and reduces conflict with competing demands.
